# Advances in LiDAR Hardware Technology: Focus on Elastic LiDAR for Solid Target Scanning

**DOI:** 10.3390/s24227268

**Published:** 2024-11-14

**Authors:** Wentao Li, Tianyun Shi, Rui Wang, Jingjie Yang, Zhen Ma, Wanpeng Zhang, Huijin Fu, Pengyue Guo

**Affiliations:** 1 Postgraduate Department, China Academy of Railway Sciences, Beijing 100081, China; lwtcon@163.com; 2 Science and Information Department, China Academy of Railway Sciences Group Co., Ltd., Beijing 100081, China; 3 Institute of Computing Technologies, China Academy of Railway Sciences Group Co., Ltd., Beijing 100081, China; 4 Railway Science & Technology Research & Development Center, China Academy of Railway Sciences Group Co., Ltd., Beijing 100081, China

**Keywords:** elastic LiDAR, laser ranging, emitters, scanning and optical modules, detectors, solid target scanning

## Abstract

This paper explores the development of elastic LiDAR technology, focusing specifically on key components relevant to solid target scanning applications. By analyzing its fundamentals and working mechanisms, the advantages of elastic LiDAR for precise measurement and environmental sensing are demonstrated. This paper emphasizes innovative advances in emitters and scanning systems, and examines the impact of optical design on performance and cost. Various ranging methods are discussed. Practical application cases of elastic LiDAR are presented, and future trends and challenges are explored. The purpose of this paper is to provide a comprehensive perspective on the technical details of elastic LiDAR, the current state of application, and future directions. All instances of “LiDAR” in this paper specifically refer to elastic LiDAR.

## 1. Introduction

LiDAR creates high-resolution point clouds of the surrounding environment by measuring the differences between emitted and reflected signals, enabling precise distance measurements to target objects [1]. LiDAR gradually expanded from the military field to the civilian market in the middle of the 20th century and has shown unique advantages and wide application potential in many fields. Its high flexibility and automation have made it essential in archaeology [2], geology [3,4], seismology [5], construction [6], and oceanography [7]. In agriculture [8,9], animal husbandry [10], and forestry [11,12], LiDAR is increasingly significant for accurate mapping and resource management. Urban applications like security monitoring [13,14] and transportation management [15,16] increasingly rely on LiDAR for intelligent surveillance. With advancing automation and digital transformation, LiDAR’s role in mining [17] and disaster warning [18] is becoming more critical. The rapid advancements in AR/VR [19], autonomous vehicles [20], and the Internet of Things [21] have expanded LiDAR’s potential as a bridge between reality and the digital world.

In the application of LiDAR technology, data processing plays a crucial role in transforming raw point cloud data into actionable insights. One of the most important steps is target detection, where algorithms analyze the point cloud to identify and classify objects within a scene. Modern deep learning techniques, such as PointNet [22] and YOLO [23], have been successfully applied to LiDAR data, enabling highly accurate and efficient detection of vehicles, pedestrians, and other objects in dynamic environments. Additionally, traditional clustering algorithms like DBSCAN [24] and regression-based methods continue to offer reliable solutions for initial object detection in point clouds.

Semantic segmentation is another critical data processing task that involves categorizing each point in the cloud according to its corresponding environment (e.g., road, building, vegetation). Techniques such as SegNet [25] and fully convolutional networks (FCNs) [26] are employed to distinguish between different categories within the scene, providing essential information for tasks like autonomous driving and robotic navigation.

Moreover, LiDAR signal processing encompasses a range of operations such as noise reduction, filtering, and enhancing the reflection signal to improve the quality of the data. Common techniques like Kalman filtering [27] and ICP (iterative closest point) [28] are used for point cloud alignment and noise suppression, which is especially important for improving data accuracy in complex environments. Additionally, advancements in machine learning and deep learning are increasingly being integrated into LiDAR signal processing, allowing for more robust data fusion when combining LiDAR with other sensor data, such as cameras or radar, to achieve more comprehensive environmental perception.

**Figure 1:**
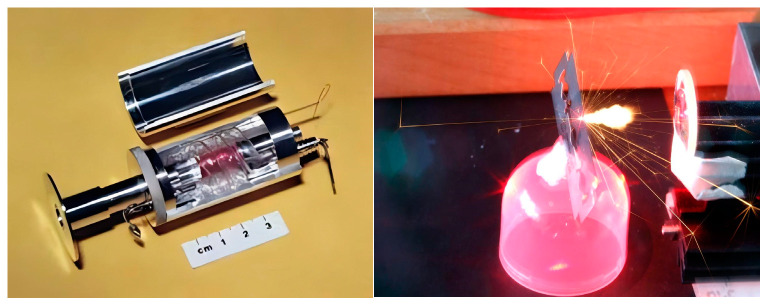
Maiman laser.

LiDAR research spans 100 years. In 1917, Einstein developed the necessary theory and formulas for constructing lasers [29], laying the theoretical foundation for the invention of lasers. In 1954, Townes and Schawlow developed the first microwave amplification by stimulated emission of radiation (MASER) based on this theory [30]. However, MASERs faced several issues: the ammonia molecules used were prone to chemical reactions and radioactive degradation, deteriorating equipment performance; MASERs operated at temperatures near absolute zero, making them difficult and expensive to operate and maintain. In 1956, Bloembergen created a MASER using silicon crystals [31], applying the principles of Townes and Schawlow [32]. Unlike ammonia molecules, silicon crystal atoms or ions emit radiation and amplify signals in the microwave range. Due to their stable performance and broader operating temperature range, silicon crystal MASERs are more widely used in microwave communication and radar systems. Since then, scientists have experimented with other solid materials to enhance performance. In 1960, Maiman developed the first ruby laser [33], marking the birth of a practical laser device, as shown in Figure 1. Maiman produced a coherent pulsed laser beam with a 694.3 nm wavelength by shining light from a flashlamp into a ruby crystal. This first laser beam met the definition and requirements of lasers: monochromaticity, coherence, and high directionality [34,35]. Although the conversion efficiency was only about 0.1%, it was a significant achievement for the time and a milestone in the history of laser technology.

**Figure 2:**
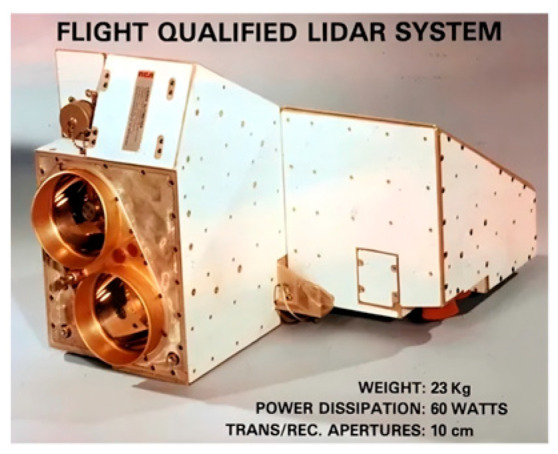
NASA’s airborne oceanographic LiDAR.

Three years after the laser’s introduction, LiDAR found its first practical application. The National Center for Atmospheric Research in the United States utilized LiDAR to study atmospheric phenomena such as cloud cover, pollution, and aerosol distribution [36]. In 1968, Hickman and Hogg developed a LiDAR-based seawater depth measurement system [37], demonstrating its potential for topographic mapping and ocean depth measurement. The lunar laser ranging experiment [38] was conducted during the Apollo 11 Moon landing in 1969. Astronauts placed mirrors on the Moon’s surface to reflect laser pulses from Earth. Measuring the round-trip time of the laser light allowed calculation of the Earth–Moon distance with centimeter-level accuracy. In the late 1970s, NASA developed an airborne oceanographic LiDAR [39], featuring scanning and high-speed data logging capabilities, capable of measuring topographic relief and bathymetry with a vertical resolution of 0.3 m, as shown in Figure 2.

Since then, airborne LiDAR has been increasingly applied to terrain surveying. In 1990, Ackermann at the University of Stuttgart, developed an airborne laser scanner for geographic mapping, integrating an instant positioning and orientation system (IPOS) [40]. IPOS includes the Global Positioning System (GPS) and an inertial measurement unit (IMU). It can obtain geographic position, attitude, and acceleration information, correct the effects of the movement in real time, and perform accurate positioning.

**Figure 3:**
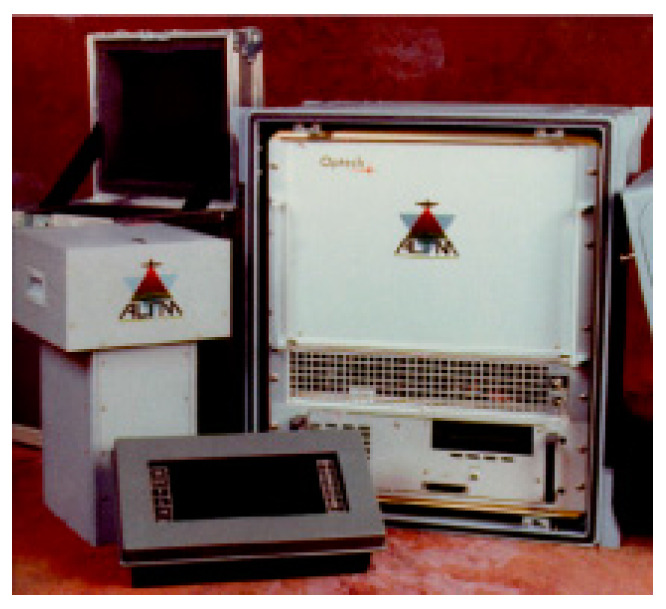
LiDAR system TopScan ALTM.

In 1993, Germany introduced the first commercially available airborne LiDAR system, TopScan ALTM 1020. Integrating GPS and IMU, it was one of the most advanced airborne LiDAR systems of its time, as shown in Figure 3.

**Figure 4:**
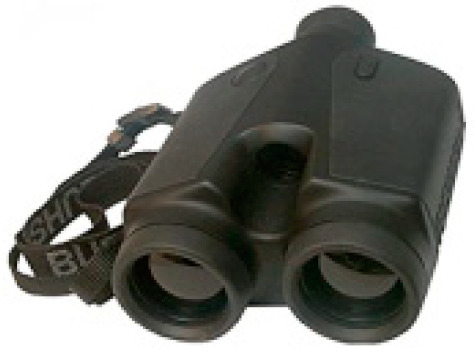
Bushnell’s Yardage Pro 400.

Between 1995 and 2000, LiDAR devices experienced significant commercial growth [41], with new systems mapping object details and salient features more efficiently. In 1996, Bushnell introduced the Yardage Pro 400 portable laser rangefinder, with a maximum range of approximately 366 m and an accuracy of ±1 yard (±0.9144 m), as shown in Figure 4. It is widely used for outdoor activities like hunting, golfing, and surveying.

In 1998, Cyra Technologies introduced the Cyrax 2500 LiDAR [42], significantly improving data collection efficiency and achieving millimeter-level accuracy within 200 m. This marked a major breakthrough in LiDAR technology. Leica Geosystems’ ALS40 and TopoSys’ Falcon series LiDAR [43] are also widely used in topographic mapping, urban planning, and forestry management.

In 2003, NASA launched a major geoscience research program, ICESat (Ice, Cloud, and Land Elevation Satellite) [44,45]. This program used satellites carrying laser altimeters called GLAS (Geoscience Laser Altimeter System) to measure changes in the polar ice surface, aiming to quantify the ice sheet’s mass balance and monitor changes. In 2005, Velodyne introduced the HDL-64E, the first commercially available, mass-produced real-time 3D LiDAR [46]. In 2007, Google used LiDAR to capture 3D environmental data, launching Google Street View [47]. In 2019, Hesai customized LiDAR for Baidu Apollo’s fifth-generation Robotaxi, increasing accuracy to 2 cm and achieving a detection range 1.5 times that of leading market products in 2020 [48]. In 2020, Apple integrated a miniature LiDAR into the iPhone 12 Pro, with a maximum range of 5 m. This improves autofocus performance, especially in low-light environments, and supports advanced AR experiences [49].

This journey reflects LiDAR’s growing maturity and diverse applications, highlighting its importance in autonomous driving, environmental capture, and consumer electronics. This paper comprehensively explores the technical details of LiDAR, including its principles, structure, ranging methods, emitters, scanning and optical systems, and detectors. It also focuses on current applications and development trends to provide an in-depth understanding of this compelling technology.

Section 2 details the basic principles and core structure of LiDAR, providing the foundation for understanding its mechanism. Section 3 covers the main ranging modes of LiDAR, providing an in-depth analysis of their principles and characteristics. Section 4 explores the types and characteristics of laser emitters. Section 5 discusses the scanning and optical systems of LiDAR. Section 6 focuses on the types and performance of detectors. Section 7 demonstrates the practical applications of LiDAR in railway foreign objects intrusion monitoring, geographic information systems (GISs) and terrain mapping, meteorological watch, and autonomous driving. Section 8 discusses the main technical challenges and future development trends. Section 9 summarizes the main points of this paper.

## 2. Working Principles and Core Components

As a vital measurement technology, LiDAR applications have expanded across various fields. An in-depth understanding of its working principles is crucial for advancing research and applications in related areas. Core components are crucial for efficient ranging, imaging, and detection tasks. Their design and performance directly impact LiDAR’s overall performance. This chapter systematically explores LiDAR’s working principles and analyzes its key mechanisms. Each core component’s working principle will also be examined for a comprehensive understanding of LiDAR.

LiDAR is a high-precision measurement system based on optical remote sensing. It calculates the distance, speed, and other attributes of a target by emitting laser signals and analyzing the differences between the emitted and reflected signals. The core components of LiDAR include the following:

(1) Emitters

Laser emitters generate laser pulses. The type, wavelength, and output power of the laser significantly impact detection distance, accuracy, and the applicable environment [50]. The primary wavelengths of emitting lasers are 905 nm and 1550 nm.

(2) Scanning and Optical Systems

LiDAR scanning systems can be categorized into conventional mechanical scanning, solid-state scanning, and hybrid solid-state scanning systems, which alter the direction of laser emission through mechanical movement or electronic control.

The optical system includes a laser emitter and various optical elements. The laser emitter collaborates with optical elements, such as lenses and mirrors, to focus and direct the laser pulse. The design and configuration of these optics determine the laser emission direction, divergence angle, and focal length, impacting measurement accuracy and range.

(3) Detectors

The detectors capture the reflected laser pulse. Typically, it is an optoelectronic sensor, such as a photomultiplier tube, photodiode, or avalanche diode, capable of converting the optical signal into an electrical signal.

(4) Receiver

The receiver refers to the entire receiving system, including the detector and electronic circuitry that amplify, filter, and process signals from the detector.

(5) Data Processing Unit

The data processing unit processes and parses signal data from the receiver, converting electrical signals into meaningful information like distance, speed, and angle [51].

## 3. Ranging Methods

Based on modulation methods, LiDAR ranging techniques can be mainly divided into time of flight (ToF), frequency modulation continuous wave (FMCW), and amplitude modulation continuous wave (AMCW).

ToF calculates distance by measuring the time difference between the transmission and reception of a laser pulse [52]. It is often referred to as direct ToF (dToF) due to the direct measurement of time difference. FMCW measures distance by analyzing changes in laser frequency, and AMCW measures the phase difference between two signals to infer the time of flight, thus FMCW and AMCW is also called indirect ToF (iToF).

### 3.1. dToF

**Figure 5:**
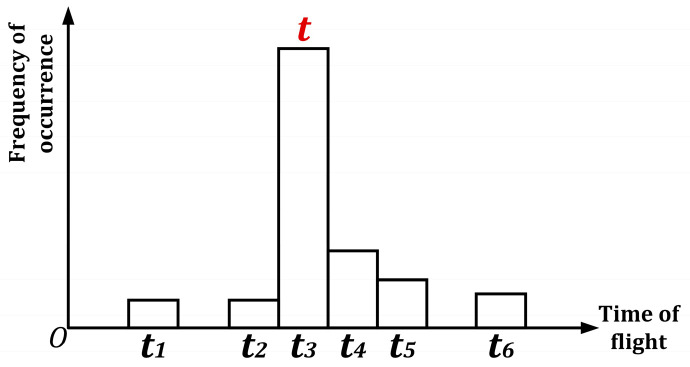
Optical signals time of flight histogram statistics based dToF.

During pulse modulation, it transmits and receives the optical signal multiple times within a single measurement frame to reduce the influence of noise and environmental factors, thereby improving data reliability. dToF is typically based on single-pulse operation, but in many implementations, histogramming is used to aggregate multiple pulse events to improve accuracy. This distance equals the speed of light multiplied by half the flight time, as shown in Figure 5.

The principle of pulse modulation is relatively simple, but achieving high accuracy is challenging. It requires high-precision clock synchronization and precise pulse signals, which ordinary photodiodes struggle to achieve. Due to the complexity of high sensitivity photodetector production, few manufacturers can handle it, making integration difficult [53].

### 3.2. FMCW

FMCW utilizes a frequency-modulated continuous-wave laser to measure the distance of an object. The laser frequency increases or decreases following a specific pattern, creating a frequency difference between the reflected and emitted light, known as the beat frequency. By measuring the beat frequency, the distance to an object is determined [54,55]. Additionally, since the frequency change is related to the relative velocity of the target object, FMCW LiDAR can directly measure the target object’s velocity.

The target depth is calculated using the following equation:

d=c∆f/2k

where *c* is the speed of light, ∆f is the frequency of the difference signal, and *k* is the rate of frequency change.

Due to its continuous wave nature, FMCW LiDAR provides higher distance resolution and better noise rejection. It achieves highly accurate distance measurements by precisely controlling the frequency change rate, allowing differentiation between multiple target objects in close proximity. Compared to pulsed radar, FMCW LiDAR can use lower peak power at the same average power, reducing reliance on high-power components and system noise.

FMCW LiDAR typically includes the following key components, which differ from those in pulsed LiDAR:

(1) Optoelectronic oscillator: Used to modulate the frequency of the laser [56].
(2) Mixer: The received signal is mixed with the principal oscillator signal (a reference signal generated internally by the radar system at a precisely controlled frequency very close to the transmitted signal) to produce an intermediate frequency (IF) signal. The IF signal is the absolute value of the frequency difference between the reflected signal and the principal oscillator signal and is usually low enough to be easily processed.

### 3.3. AMCW

**Figure 6:**
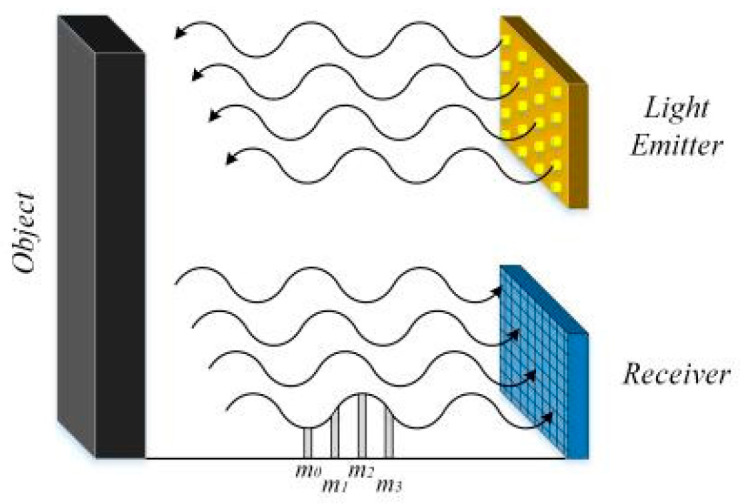
Schematic diagram of AMCW imaging principle.

AMCW indirectly calculates the time of flight by measuring the phase offsets of the transmitted and received signals, rather than directly measuring the time of flight. The phase shift is calculated based on the accumulated charge during the exposure (integration) time [57], as illustrated in Figure 6.

The depth information of each object in the scene is derived from the phase offset data. A widely used method for calculating depth is the four-sampling-bucket algorithm [58], which utilizes four sampling signals with different phase delays to calculate depth.

The target depth is calculated using the following equation:

$$d=c∆\varphi /2\pi f_{m}$$

where *c* is the speed of light, ∆φ is the phase difference, and $f_{m}$ is the modulation frequency.

### 3.4. Performance Comparison

Basic evaluation metrics for ranging technology include ranging accuracy, effective detection distance, and image resolution. Additionally, when integrating into 3D applications like mobile terminals, energy consumption, cost, and interference immunity in complex scenarios must be considered. These specifications dictate that dToF and iToF have distinct application scenarios.

(1) Accuracy

Accuracy refers to the difference between the true depth value and the measured value. The theoretical accuracy of dToF can reach 10^−12^ s × 3 × 10^8^ m/s = 3 mm. Due to the presence of quantum noise [59] and amplifier noise [60] in the avalanche process, and inherent noise in the time-to-digital converter [61], the practical accuracy of current dToF systems can only reach the cm level, similar to iToF. However, theoretically, the measurement error of dToF does not increase with distance. iToF performance may be affected by distance, ambient light, and surface reflection characteristics. dToF technology still holds advantages in long-distance measurement and high-precision demand scenarios.

(2) Effective Detection Distance

The effective detection range of an iToF system is constrained by modulation frequency, signal-to-noise ratio, ambient light interference, and reflectivity, making it suitable for shorter distances (tens of meters). dToF systems, relying on high-precision time measurements rather than phase changes, typically achieve much longer detection ranges.

(3) Power Consumption

dToF emits short, pulsed lasers with ns-level or even ps-level durations, while iToF uses continuous wave modulation. Pulsed waves achieve ultra-low duty cycles and thus lower power consumption. Additionally, since the measurement accuracy of dToF does not decrease with distance, it consumes less power for long-distance measurements. Conversely, iToF uses surface light emission. As measurement distance increases, iToF needs to increase light power or extend exposure time to achieve higher accuracy, significantly increasing power consumption.

(4) Anti-interference

dToF acquires distance information through multiple measurements and uses histogram statistics to calculate the flight time, making it easier to distinguish interference signals. iToF calculates the phase shift with mixed ambient and modulated light, making it difficult to distinguish interference. The stronger the ambient light, the greater the error caused.

(5) Application Scenario

With low power consumption and small size, dToF is suitable for applications like robotics that require fast ranging and obstacle avoidance, as well as for compact designs with space constraints. It exhibits higher accuracy and interference immunity in outdoor scenarios.

Despite the shorter detection distance, the higher resolution of iToF images can reproduce more detailed information in applications such as object recognition, 3D reconstruction, and behavioral analysis.

## 4. Laser Emitters

### 4.1. Chip Types

Common types of lasers currently include laser diodes (LDs), edge emitting lasers (EELs), vertical cavity surface emitting lasers (VCSELs), solid-state lasers (SSLs), fiber lasers (FLs), gas lasers (GLs), and dye lasers (DLs).

#### 4.1.1. LD

**Figure 7:**
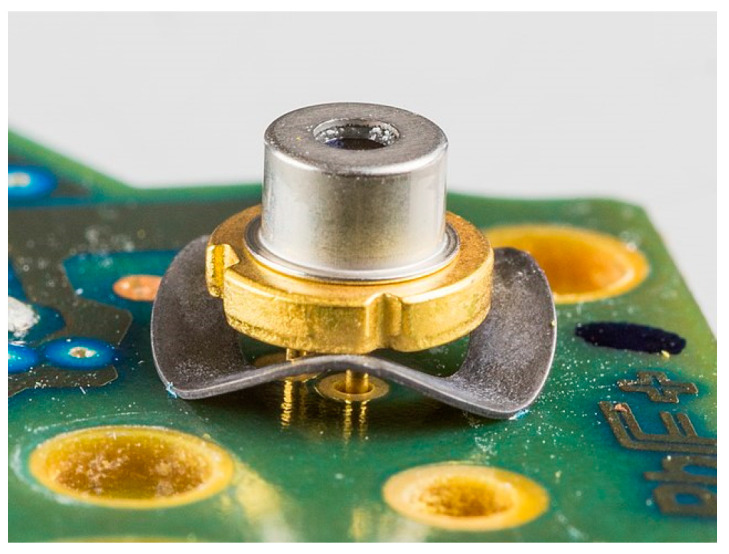
The LD of a LiDAR.

LDs are light sources based on semiconductor materials and form the foundation of modern laser technology, the LD of a lidar is shown in Figure 7. In 1962, Hall at Bell Labs developed the first practical LD [62]. This laser used GaAs as the semiconductor material and had a wavelength of 850 nm. Initially, the device only worked in pulse mode and needed to be cooled to liquid nitrogen temperatures (77 K). Significant improvements in the design and fabrication of LDs were made over the next few years, including the introduction of the double heterostructure [63], which increased laser’s generation efficiency and output power. LDs began transitioning from the laboratory to commercialization.

In 1965, Holonyak Jr. developed the first red GaAsP laser in the visible spectrum [64], marking a major breakthrough in LDs. In the 1970s, researchers developed new semiconductor materials, such as InGaAsP and AlGaAs. The introduction of these materials enabled LDs to operate over a wider wavelength range and stably at room temperature. In the 1980s, quantum-well lasers entered mass production, increasing laser efficiency and performance while lowering the threshold current, allowing lower currents to excite the laser. In the 1990s, LDs made significant progress in high power output and tunable wavelengths. This was mainly due to the development of quantum-well and quantum-dot technologies, as well as improvements in materials and design, enhancing their importance in fiber optic communications, especially in wavelength-division multiplexing (WDM) systems. In the 21st century, LD research has focused on developing photonic integrated circuits and multiwavelength lasers. These technologies have enabled LDs to be used in more complex systems [65]. In recent years, advances in nanotechnology and micromachining have further reduced the size of LDs and improved their performance, opening new avenues for applications in emerging fields such as medical imaging, sensing, and quantum communications. Kyoto University has invented a new type of LD expected to revolutionize ranging from LiDAR to manufacturing [66]. The key innovation is its use of photonic crystals that emit a circular beam of high brightness and low divergence (as small as 0.1 degrees) vertically from the top.

#### 4.1.2. EEL

**Figure 8:**
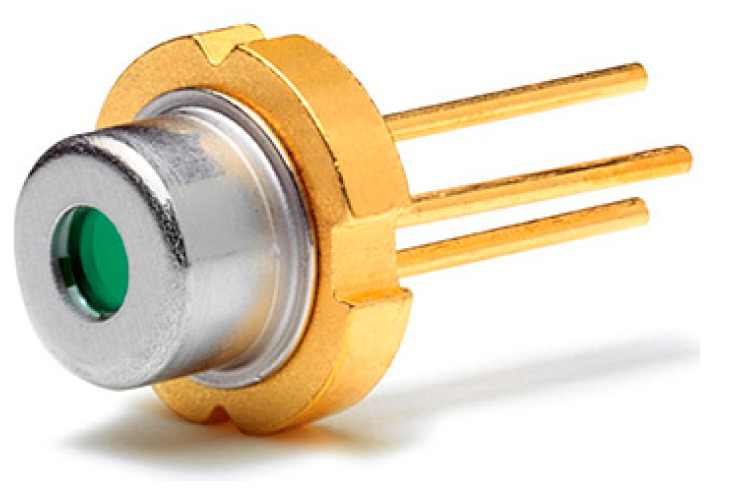
The EEL of a LiDAR.

As a type of LD, EELs inherit many characteristics of semiconductor lasers. EEL usually adopts a planar waveguide structure in which the laser propagates along the edge of the waveguide and emits light from the end of the waveguide, the EEL of a LiDAR is shown in Figure 8.

The development of EELs began in the early 1960s with the generation of light by passing an electric current through a semiconductor material. During the 1970s, EELs saw gradual improvements in performance, including increased optical output power and reduced spectral widths, making them more useful in telecommunications and fiber optic communication. In the 1990s, EELs became smaller in size and power consumption, becoming the primary laser source in fiber optic communication systems. Currently, EELs are widely used in communication, laser printing, LiDAR, and medical devices. Their performance and reliability are constantly improving, playing an important role in optoelectronics.

#### 4.1.3. VCSEL

**Figure 9:**
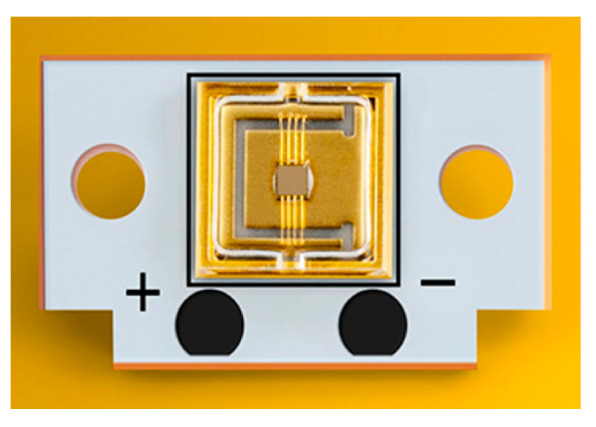
The VCSEL of a LiDAR.

VCSELs, although different from EELs, are also LDs and represent another form of LD technology, the VCSEL of a LiDAR is shown in Figure 9.

In the 1990s, VCSEL technology transitioned from laboratory to industrial production, and the first commercial VCSEL products were introduced [67], mainly used in data communication, such as fiber-optic communication. In 2000, VCSEL technology saw significant performance improvements, including increased power output and efficiency, allowing for use in a wider range of applications, such as consumer electronics and medical imaging. In 2010, significant breakthroughs were made in areas like 3D sensing and facial recognition, with the widespread use of smartphones driving rapid VCSEL market growth [68]. By 2020, VCSEL technology continued to evolve, with research focusing on improving beam quality, increasing wavelength range, and reducing costs to meet the needs of advanced applications such as autonomous driving and high-speed data center communications.

#### 4.1.4. SSL

SSLs have played a significant role in the history of laser technology. In the 1960s, the concept of SSLs was first proposed and realized, ushering in a new era of laser technology. In the 1980s, technological advances in SSLs included improvements in wavelength tuning and pulse operation, leading to a wide range of applications in the medical field. In the 1990s, SSL development focused on downsizing and improving system integration to accommodate portable and consumer electronics. In the 2020s, SSL technology continues to evolve toward higher power [69], a wider wavelength range, and better beam quality to meet growing market demand.

#### 4.1.5. FL

**Figure 10:**
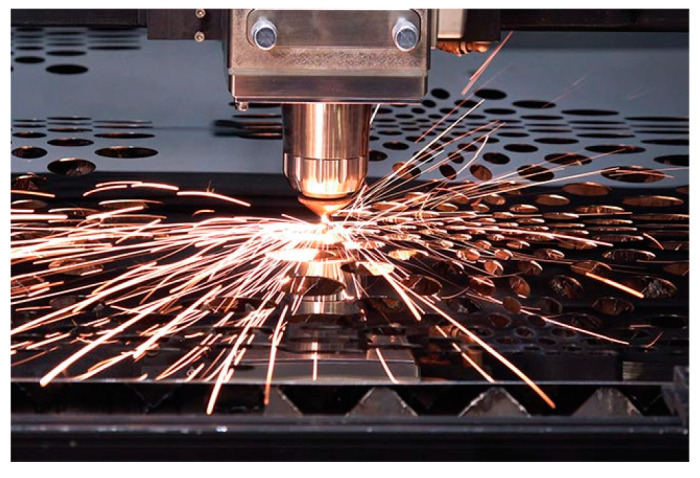
The FL is cutting metal.

FLs complement SSLs in many applications and represent a significant development, the FL of a LiDAR is shown in Figure 10. The concept of FL was first introduced in the 1960s [70]. In the 1990s, significant improvements in output power and efficiency made it feasible for industrial processing and medical therapy. In the 2010s, FLs dominated advanced manufacturing for their high power, efficiency, and beam quality, especially in metal cutting and welding [71].

In the 2020s, the focus of FL research has shifted towards further increasing power density, reducing costs, and achieving greater functional integration. Ultra-high power FLs now exceed 40 kW, significantly improving cutting speed and accuracy, particularly in thick metal cutting applications [72]. Additionally, improvements in electro-optical conversion efficiency have markedly reduced the operating costs of FLs. Modern FLs also incorporate tunable spot shapes, zoom optical heads, and mixed gas cutting technologies, enhancing their flexibility and efficiency in handling various materials and thicknesses [73]. The integration of machine learning and AI has further enhanced the performance and reliability of FLs [74].

#### 4.1.6. GLs

**Figure 11:**
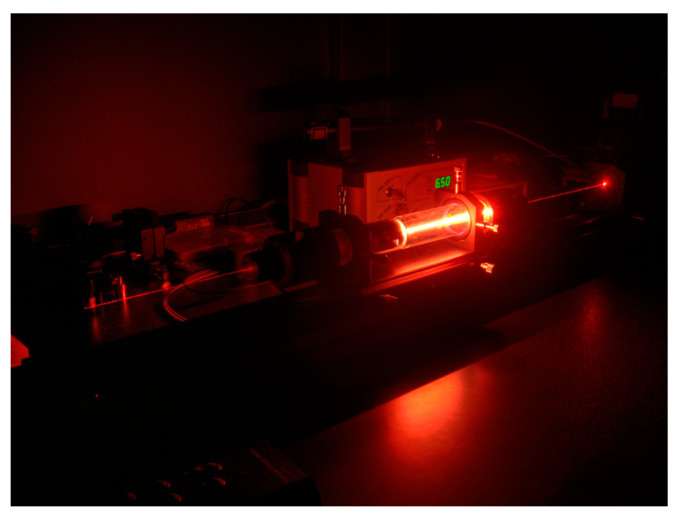
A helium–neon laser.

GLs are significant representatives of early laser technology; a GL is shown in Figure 11. In the 1970s, GLs advanced in output power and wavelength tuning, with CO_2_ lasers particularly used in industrial processing [75]. In the 2000s, GLs made important advances in ultrafast pulse generation and precision machining [76,77]. By the 2020s, GL research and development focused on further improving performance, reducing costs, and exploring new application possibilities.

#### 4.1.7. DLs

**Figure 12:**
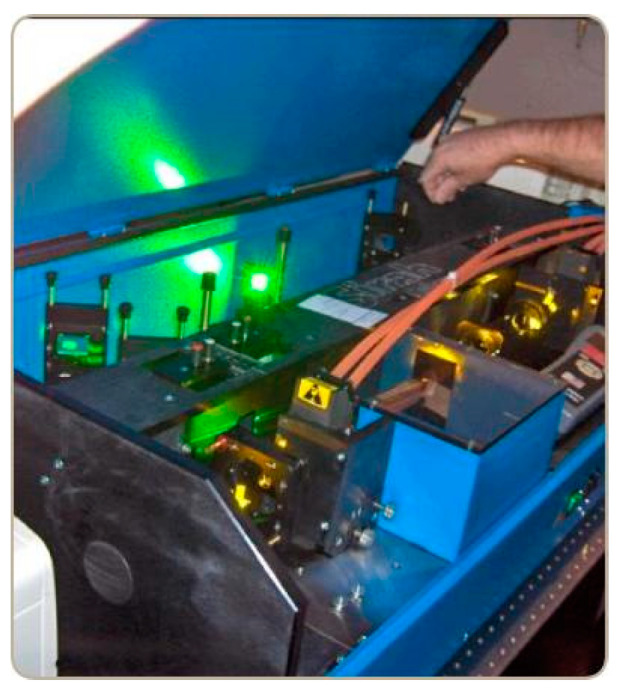
The Sirah DL undergoing adjustments.

DLs use organic dyes as the lasing medium and can operate over a wider range of wavelengths than GLs or SSLs; a DL is shown in Figure 12. DLs were independently discovered by Sorokin [78] and Schäfer in 1966. In the 1980s, DLs found important applications in spectroscopy and nonlinear optics. In the 1990s, DLs saw improvements in efficiency and stability through advancements in dyes and pumping techniques. In the 2000s, despite rapid advances in SSLs and LDs, DLs retained their importance in specific applications, especially in ultrashort pulse generation [79]. In the 2010s, although DLs faced competition from more modern laser technologies, they remained irreplaceable in certain high-precision applications, such as high-resolution spectroscopy.

### 4.2. Wavelength

**Figure 13:**
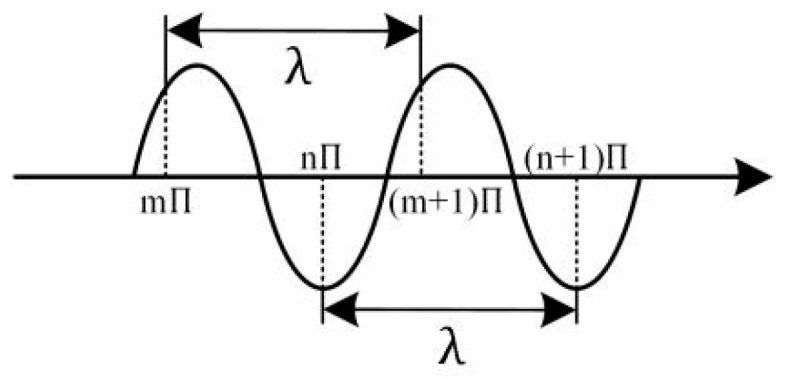
Wavelength.

A laser is essentially an electromagnetic wave characterized by wavelength and intensity. The wavelength refers to the distance an electromagnetic wave propagates in one vibration cycle, i.e., the distance between two neighboring points with a 2π difference in vibrational phase along the propagation direction, as shown by λ in Figure 13.

**Figure 14:**
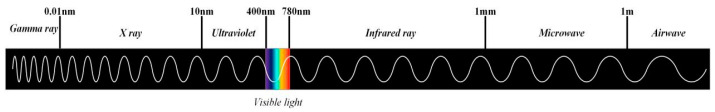
Electromagnetic spectrum.

Figure 14 illustrates the electromagnetic spectrum.

Visible light, which ranges from approximately 400 to 780 nm in wavelength, is the portion of the electromagnetic spectrum visible to the human eye. Currently, Lidar systems primarily utilize laser sources with wavelengths of 905 and 1550 nm, both of which fall within the infrared spectrum (>750 nm). This preference is due to two main reasons:

(1) Personal safety: Gamma rays, X-rays, ultraviolet rays, and visible light can harm humans and the environment, whereas infrared rays are relatively safe.
(2) Higher spatial resolution: Infrared wavelengths are shorter than those of microwaves or radio waves, providing finer images and data. This makes infrared ideal for applications requiring the detection of smaller objects and accurate ranging.

#### 4.2.1. 905 nm

Being in the near-infrared spectral region, 905 nm is relatively safe for the human eye, contributing to its widespread use. Additionally, lasers at 905 nm can be detected by standard silicon photodetectors, helping to control costs [80,81,82,83]. With the advent of LDs, 905 nm LiDAR has become smaller, lighter, and more cost effective, facilitating their application across various fields.

#### 4.2.2. 1550 nm

The 1550 nm wavelength is in the mid-infrared spectral region, which is safer for the human eye, reducing potential eye damage. However, unlike the 905 nm wavelength, 1550 nm lasers usually require InGaAs photodetectors instead of standard silicon photodetectors, increasing the cost [84,85]. Despite this, 1550 nm LiDAR is widely used for long-range and high-accuracy measurements due to its high safety.

#### 4.2.3. Performance Comparison

(1) Detection Distance

The maximum detection range of 905 nm LiDAR at 10% reflectivity is typically up to 200 m. The 1550 nm laser has a more relaxed power limit than the 905 nm laser. Consequently, it can emit laser beams at higher power, achieving longer detection distances and greater anti-interference capabilities.

(2) Industry Maturity and Costs

The 905 nm LiDAR detector uses traditional silicon-based materials, compatible with the existing silicon industry chain. This mature technology is easy to produce, with lower development difficulty and cost.

Silicon detectors are sensitive to visible to near-infrared light (about 400 to 1100 nm), but their optical absorption decreases rapidly at longer wavelengths (e.g., 1550 nm), resulting in lower detection efficiency. 1550 nm laser detectors typically use InGaAs, which are expensive and less mature than silicon-based materials.

Additionally, 1550 nm lasers are generally FLs, which are complex systems with several precision components. The larger size and higher heat generation are reasons why most 1550 nm LiDAR have only one laser. To achieve sufficient field of view (FOV) and point density, 1550 nm LiDAR often uses a 2D rotating mirror, involving more internal moving parts. Ensuring the life and stability of these parts requires significant research costs.

(3) Power and Heat Dissipation

The emission power upper limit of a 1550 nm laser is 40 times higher than that of 905 nm [86,87]. Since 905 nm LiDAR has less power, heat dissipation is less demanding, making heat management easier and potentially extending its lifespan. Furthermore, 905 nm LiDAR typically employs multiple lasers, whereas 1550 nm LiDAR generally has only one. Consequently, the load on each 905 nm laser is much smaller than on 1550 nm. Multiple lasers share the scanning task, reducing the workload per laser and helping to extend their lifetime. The larger number of lasers also provides some robustness.

(4) Penetrating

Laser propagating in the environment is affected by various types of suspended solids. According to wave diffraction phenomena, the larger the wavelength, the better the evasion performance. When the size of the suspended solids is much larger than the wavelength, the laser usually cannot bypass it.

For example, the average diameter of haze particles is about 1000 to 2000 nm [88]. Therefore, the penetration ability of the 1550 nm laser is significantly better than that of 905 nm on hazy days. The average diameter of fog particles is about 10,000 to 20,000 nm, which is much larger than the wavelength, meaning both 905 and 1550 nm lasers do not perform well in foggy weather.

(5) Divergence Angle

**Figure 15:**
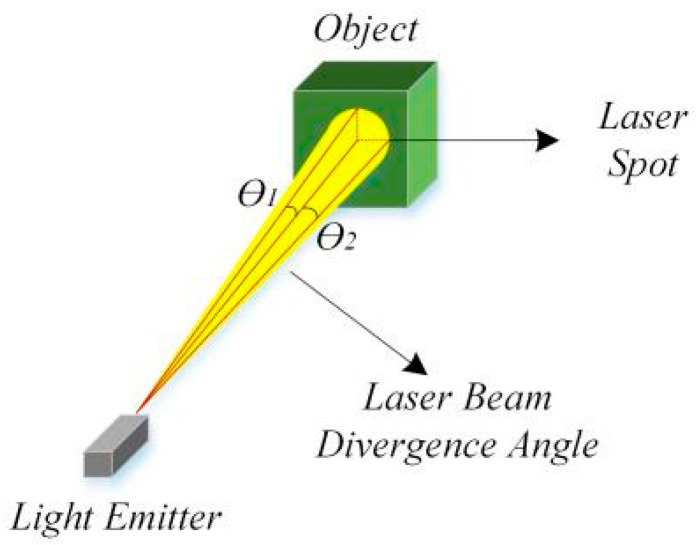
Laser beam divergence angle and spot.

The divergence angle is the angle range at which a laser beam spreads after leaving the emission source. A smaller divergence angle indicates a more focused beam, maintaining higher energy density over longer distances. For instance, if a laser beam has a divergence angle of 0.1° × 0.1°, its spot size will be approximately 17 cm × 17 cm at 100 m, as shown in Figure 15.

Due to the large area of this spot, when the projected area of the measured object is smaller than the spot, or when the laser hits the edge of the object, multiple reflections at different distances will occur. This results in LiDAR receiving multiple echoes, leading to measurement errors. A smaller divergence angle means higher resolution at longer distances. Therefore, a smaller divergence angle is preferable, with the spot being small enough.

The divergence angle is affected by various factors, including the emitter, optical path, power, and wavelength. Assuming the emitter, power, and optical path are the same, a longer wavelength results in a larger divergence angle. Therefore, a 1550 nm wavelength laser beam theoretically has a relatively larger spot. However, for current vehicle LiDAR, 1550 nm is mostly used for FLs, while 905 nm is mostly used for LDs. FLs have a smaller exit spot, advantageous for long-distance detection. LDs require a larger optical system to achieve a similar spot size as FLs, which is challenging in miniaturized applications.

(6) Water Absorption Rate

Water affects the integrity of the laser signal. In rain and fog, 1550 nm laser light experiences four to five times higher attenuation levels than 905 nm, and more than 90% higher signal attenuation in snow. When reflecting off water and ice-covered surfaces, the reflectivity of 1550 nm laser signals decreases by about 60%, while 905 nm decreases by 15% [89]. Therefore, the National Oceanic and Atmospheric Administration, in its report, does not recommend using 1550 nm LiDAR in wet conditions.

**Figure 16:**
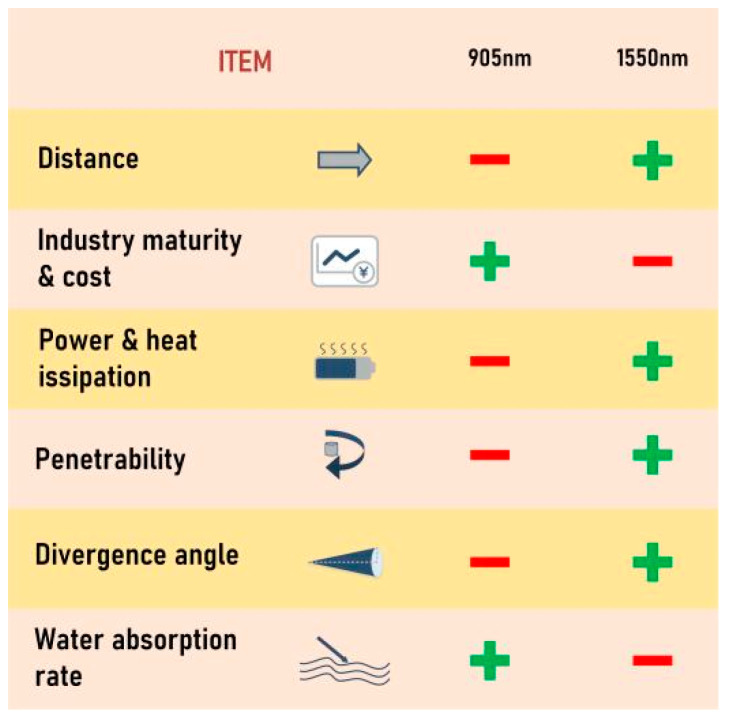
Comparison of LiDAR performance at 905 and 1550 nm wavelengths.

The conclusion of the LiDAR performance comparison between 905 and 1550 nm wavelengths is shown in Figure 16. The “+” symbol indicates higher performance, while the “−” symbol indicates lower performance.

## 5. Scanning and Optical Systems

The current trend in LiDAR is the gradual replacement of mechanical LiDAR (mech LiDAR) by solid-state LiDAR. Solid-state LiDAR (solid LiDAR) is promising for various applications due to its high resolution, reliability, and adaptability [90]. The primary difference between mechanical, solid-state, and hybrid solid-state LiDAR (hybrid LiDAR) lies in their scanning and optical systems.

### 5.1. Mech LiDAR

Mech LiDAR uses rotating lenses or assemblies to scan the environment. This design offers good detection range and resolution but is prone to wear and tear and is large and costly due to its mechanical moving parts.

The concept of mech LiDAR began to take shape in the 1960s with the invention of the laser. Initial mech LiDARs were large, expensive, and had limited data accuracy, but their potential was obvious, prompting scientists to improve their accuracy and performance [91]. In 1971, mech laser altimetry was first used to measure the lunar surface during the Apollo 15 mission, with the primary objective of making precise measurements of lunar surface elevation [92,93]. This was the first application of mech LiDAR in space exploration, providing technical reference and experience for subsequent missions. In the 1980s, mech LiDAR was primarily used in earth science [94] and atmospheric science [95] to measure atmospheric composition and cloud heights. Simultaneously, it was introduced into ground mapping [96] and GIS [97] to obtain elevation and shape information on the Earth’s surface. In the 1990s, mech LiDAR was widely used in the military for detecting and analyzing targets [98], as well as for collision avoidance, landing assistance, and terrain mapping with military UAVs [99]. From 2010 to the present, mech LiDAR research has focused on higher resolution, longer range, and faster scanning speeds. Additionally, researchers have focused on integrating mech LiDAR with other sensors to provide multi-layered environmental sensing and mitigate the limitations of individual sensors [100,101]. Researchers have also focused on overcoming adverse weather [102]. Currently, the development of mech LiDAR focuses on enhancing detection capabilities in complex and dynamic environments and strengthening integration with other sensors to improve the comprehensiveness and robustness of environmental sensing. Additionally, efforts to realize the intelligence and automation of the sensing system are crucial for promoting its application in fields such as smart cities and UAV surveillance.

### 5.2. Solid LiDAR

Solid LiDAR eliminates mechanical components for environmental scanning, using optical phased arrays (OPA) or other non-mechanical techniques to manipulate laser beams [103]. Consequently, these systems are inherently more robust and dependable, often characterized by a compact form factor and reduced weight.

#### 5.2.1. OPA LiDAR

**Figure 17:**
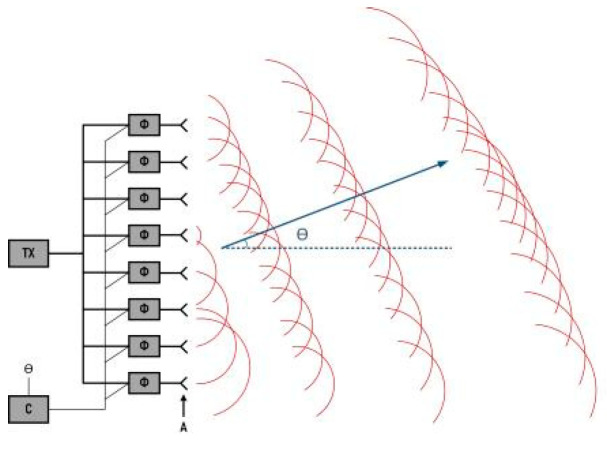
Phased array schematic.

OPAs use electronically tunable phase modulators to steer the beam direction. These arrays consist of miniature optical elements, each capable of phase tuning to alter the beam’s trajectory [104]. The origin of LiDAR technology using OPAs can be traced to phased array systems, representing an adaptation of active phased array principles to the optical spectrum. Phased arrays manipulate the phase and amplitude of individual electromagnetic wave components to concentrate wave intensity in a desired direction while diminishing it elsewhere. This directional control effectively steers the beam. A visual representation of phased array operation is depicted in Figure 17.

Active phased arrays are a specialized variant of phased arrays, where each antenna element has independent phase control and its own transmitter–receiver module. This configuration allows each antenna to independently amplify or receive signals, enhancing flexibility and performance. Microwave phased arrays, operating in the microwave frequency band, are the first generation of active phased array systems to achieve practical application. Since the first implementation of active phased arrays in 1937 [105], this technology has been extensively used in both civilian and military domains.

OPAs and microwave phased arrays share a fundamental operating principle. However, the high frequency and short wavelength of light waves, along with the lack of efficient phase modulation methods for optical fields, have impeded the advancement of OPAs. The scarcity of devices capable of modulating the optical phase has constrained the development of OPA. The 1D OPA concept was first realized by Meyer in 1972, using a LiTaO_3_ crystal phase shifter [106]. This pioneering work validated the feasibility of OPAs and sparked a surge in research over the following decades [107]. The emergence of silicon photonics at the turn of the century provided a new platform for advancing OPA. In 2013, Sun showcased the fabrication of large-scale nanophotonic phased arrays using silicon photonics [108], marking a significant milestone for high-performance, cost-effective LiDAR. Further progress was made in 2020 when Abiri presented an integrated OPA with over 512 elements, representing a substantial leap in scale and integration for LiDAR-specific silicon photonic phased array technology.

However, silicon-based OPAs face several technical hurdles, such as controlling the beam divergence angle, managing the laser power threshold for silicon waveguides, and optimizing chip power consumption. The current trajectory of OPA LiDAR technology involves overcoming these challenges while enhancing performance metrics, such as increasing the scan rate and expanding the detection range. Looking ahead, the evolution of this technology will focus on enhancing reliability, reducing costs, and fine-tuning performance to meet the demands of autonomous driving and other emerging applications.

#### 5.2.2. Flash LiDAR

Flash LiDAR emits a broad beam of laser pulses instantaneously across its entire FOV, illuminating the scene. These pulses reflect off object surfaces and return to the receiver. By analyzing the time delay and intensity of the reflected light, the system rapidly generates a 3D representation of the scene.

In its early stages, flash LiDAR was predominantly used in space and aviation applications. NASA used a 3D imaging flash LiDAR for the automated landing of spacecraft on planetary bodies [109,110]. NASA continued to refine the technology and algorithms, enhancing the spatial resolution, range, and photometric precision of 3D LiDAR imagery [111]. The MIT Media Lab explored the bounce-flash LiDAR, a novel variant of flash LiDAR offering enhanced flexibility in scene illumination [112].

The integration of advanced light source and detector technologies in modern flash LiDAR has led to significant improvements in luminosity accuracy and detection range. These advancements enable the generation of more precise and extensive 3D images. Moreover, the enhancement of data processing algorithms has substantially increased the speed and accuracy of 3D image construction. Sense Photonics has introduced the world’s first 940 nm global shutter flash LiDAR for the large-scale automotive market, capable of detecting a 10% reflectivity target at 200 m in full daylight [113]. RoboSense introduced its latest M3 LiDAR. Using a 940 nm laser, this product has ultra-long distance measurement capability and high resolution, can detect traffic cones at 270 m, and supports L3 autonomous driving at speeds of up to 120 km/h [114].

However, flash LiDAR still faces some challenges. Since its working principle requires the transmission and reception of a large number of optical signals, this puts high demands on hardware design, especially in terms of power consumption and thermal management of emitters and detectors. In addition, the performance of flash LiDAR may be affected to some extent under bright light irradiation and bad weather, which requires further technical improvements to enhance its environmental adaptability.

### 5.3. Hybrid LiDAR

Hybrid LiDAR integrates mechanical components with solid-state technology, leveraging the stability and reliability of the latter while harnessing the high accuracy and resolution of mech LiDARs. This approach aims to provide an expansive scanning range while reducing mechanical wear and tear. Mechanical elements, such as rotating mirrors or galvanometers, are commonly used in scanning systems to enable wide-angle environmental scans. In contrast, solid-state technologies are predominantly used for emitters and detectors, with solid-state lasers serving as light sources and photodetectors capturing reflected signals. The advent of hybrid LiDAR around the turn of the century marked a pivotal step in LiDAR technology, steering it towards enhanced integration, reliability, and cost-effectiveness.

Hybrid LiDAR can be differentiated by their scanning methodologies, classified into one-dimensional and two-dimensional scanning types. The two-dimensional scanning category includes rotary mirror, prismatic, and MEMS microscanner technologies.

#### 5.3.1. Rotating Mirror Style

Rotating mirror style LiDARs use one or more rotate mirrors to dynamically alter the laser beam’s trajectory, achieving a high resolution and extended scanning range. This design offers precise control and superior angular resolution. However, it also introduces challenges such as slower scanning speeds and increased susceptibility to wear and mechanical failure.

#### 5.3.2. Prism Style

Prism LiDARs deflect a laser beam using one or more stationary prisms, scanning the environment by adjusting the angle of incidence. This approach is often integrated with solid-state technology to bolster the system’s durability and reliability. The use of fixed prisms eliminates the need for moving parts, reducing potential mechanical failure and extending the system’s lifespan.

#### 5.3.3. Rotating Mirror and Prism Style

**Figure 18:**
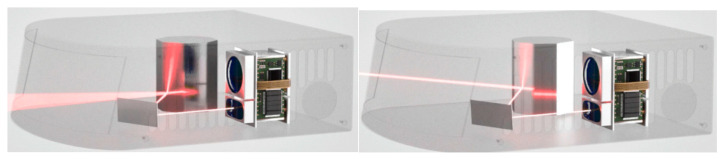
Rotate mirror + prism scanning method.

This hybrid LiDAR uses a polygonal prism that rotates continuously around a horizontal axis and a rotate mirror that pivots along a vertical axis. The following is a 2D rotating mirror scanning diagram available on the market. The constantly rotating polygon prism can make the light source scan horizontally, while the vertical axis of the rotating mirror can change the vertical scanning direction of the light source. The method of rotating mirror and prism scanning is shown in Figure 18.

#### 5.3.4. Micro Electromechanical Systems (MEMS) Style

MEMS is a technology that integrates tiny mechanical components, sensors, actuators, and electronic components on a silicon substrate, usually manufactured using a micromachining process. MEMS microgalvanometer LiDAR use micromachines to precisely direct the laser beam’s scanning path and determine the distance and position of objects by capturing the reflected light.

**Figure 19:**
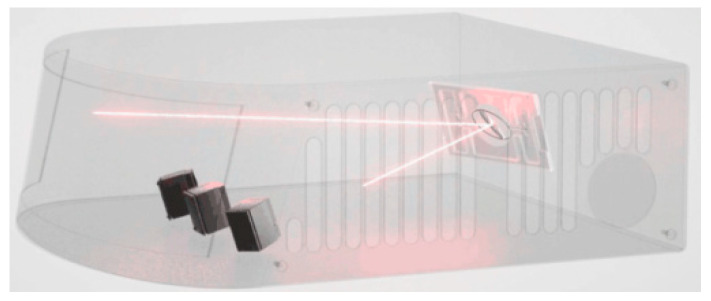
MEMS scanning method.

At the core of the MEMS LiDAR is a centimeter-sized galvanometer, actuated by a cantilever beam oscillating rapidly along the horizontal and vertical axes. This high-speed motion redirects the laser’s reflection, enabling scanning, as illustrated in Figure 19. The MEMS system streamlines the scanning architecture compared to traditional mech LiDAR. By adjusting the micro-oscillator’s deflection angle, it can alter the scanning path. Moreover, it requires fewer lasers to match the coverage area and point cloud density of a multi-beam mech LiDAR.

However, the MEMS scheme faces challenges due to the cantilever beam’s limited rotation angle, restricting the single galvanometer’s FOV. To attain a wider FOV, multiple scans must be stitched together, which can result in uneven distortion and overlap at the point cloud image edges during superposition. This also heightens the complexity of subsequent algorithmic processing.

## 6. Detector

The detector captures reflected laser pulses and converts the incoming optical signals into electrical ones. This conversion is essential for subsequent processing and analysis, allowing the system to accurately determine distances and create high-resolution images.

### 6.1. Photomultiplier Tube (PMT)

**Figure 20:**
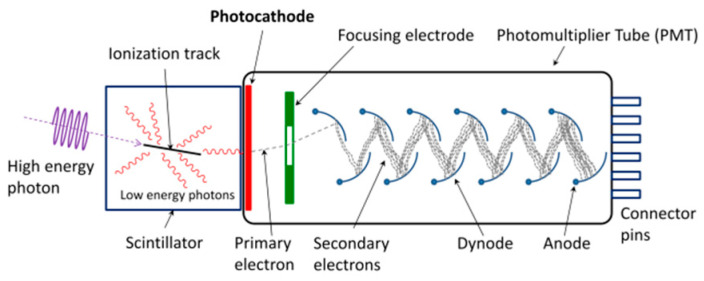
Photomultiplier tube working principle.

PMTs are known for high speed, low noise, and high gain, capable of detecting even single photons, as depicted in Figure 20. The first PMT was developed by Iams and Salzberg [115]. Historically significant in LiDAR, PMTs were initially used for meteorological observations and topographic mapping. For example, Hegde used a PMT-based LiDAR for meteorological studies [116].

With technological progress, semiconductor detectors are increasingly replacing PMTs in modern LiDAR. Their compact size, efficiency, and cost-effectiveness make them particularly suitable for portable and vehicle-mounted applications. Nonetheless, PMTs remain relevant in scientific applications demanding ultra-high sensitivity [117,118].

### 6.2. PIN Photodiodes

A photodiode is a light-sensitive device that converts photons into electrical current or voltage signals. Common solar panels use numerous photodiodes to generate electricity. In the early 1950s, standard photodiodes were tested in LiDAR; however, they lacked the necessary response speed and sensitivity.

The 1960s saw significant advancement with the introduction of the PIN photodiode. PIN photodiodes offer improved response times and higher sensitivity compared to earlier models. While cost-effective and suitable for some low-performance LiDAR applications, PIN photodiodes have largely been replaced by more sensitive and faster detectors.

### 6.3. Avalanche Photodiode (APD)

**Figure 21:**
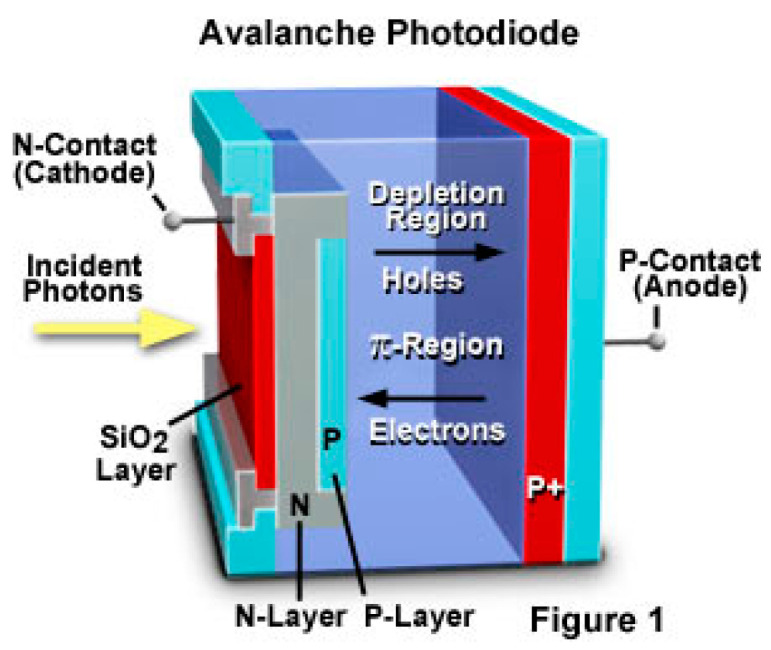
APD working principle.

APD is a highly sensitive photodetector that uses the avalanche multiplication effect in semiconductor materials to amplify the photocurrent generated by incident light, as shown in Figure 21. In the 1970s, APDs emerged as an ideal choice for laser signal reception due to their high gain and low noise characteristics. By the 1990s, significant advancements in manufacturing technology had enhanced APD performance and substantially reduced their costs. During this period, researchers focused on miniaturizing APDs to fit the compact designs of modern LiDAR.

In the 2010s, APD development was further propelled by sophisticated semiconductor processes and innovative optoelectronic device designs. Ongoing research aims to enhance APD performance by boosting quantum efficiency, response speed, and linear dynamic range. These improvements are crucial to meet the stringent requirements of high-resolution, long-range, high-accuracy LiDARs. With the evolution of fiber optic communication technology, APDs are increasingly integrated into LiDAR communication systems, expanding their application spectrum.

### 6.4. Single-Photon Avalanche Diode (SPAD)

**Figure 22:**
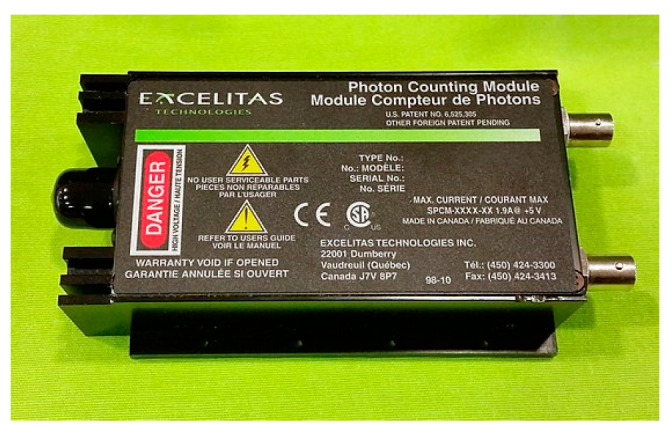
A SPAD module.

An SPAD is a distinctive avalanche photodiode that detects and amplifies single photon events. Once a photon enters an SPAD and generates an electron-hole pair, this pair of carriers initiates an avalanche effect under the influence of a strong electric field, resulting in an instantaneous increase in the current to a detectable magnitude. A SPAD module is shown in Figure 22.

SPADs are known for their digital output, single-photon sensitivity, rapid gating capabilities, and picosecond timing accuracy. These attributes, along with their compact size, cost-effectiveness, and ease of integration, make SPADs ideal for reconstructing intensity- and time-dependent waveforms from weak light signals.

Initially, SPADs found their niche in academic research areas like astrophysics, adaptive optics, and fluorescence imaging. Over time, the commercial and industrial potential of these detectors has grown, especially with the advent of smart single photon counting and time-correlated single photon counting integrated on-chip. This development has attracted major corporations like Toyota, Sony, Samsung, and Apple, especially in the mobile and consumer electronics sectors. Today, SPADs are used in various fields, including biophotonics, LiDAR, 3D optical ranging, and quantum information technology, showcasing their versatility and impact in modern technology. In radiation detection, SPAD is increasingly replacing traditional PMTs.

### 6.5. Silicon Photomultiplier (SiPM)

**Figure 23:**
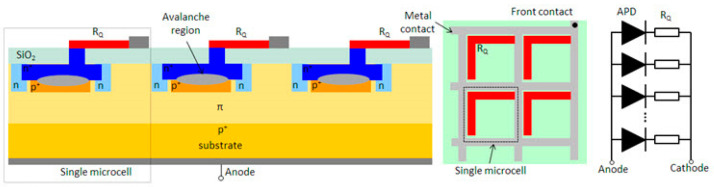
The basic model of an SiPM.

SiPM is a silicon-based photodetector characterized by an extremely high photon detection sensitivity, the basic model of an SiPM is shown in Figure 23. It comprises multiple arrays of SPADs, with each SPAD unit being capable of independently detecting a single photon and generating an electrical signal. When a photon impinges upon the SiPM, it triggers the avalanche effect of a single SPAD, resulting in the generation of an observable pulse of current. SiPM is capable of multiplying and enhancing optical signals by summarizing the output signals of multiple SPAD units.

SiPMs first appeared in the LiDAR field in the 1990s, noted for their high gain, low noise, and single-photon resolution capabilities. Early research focused on refining the fabrication process and enhancing SiPM stability to meet the stringent requirements of LiDAR, leading to their use in distance measurement and target detection. From 2010 to 2020, SiPM application in LiDAR expanded significantly, driven by technological advancements that improved spectral response, quantum efficiency, and response speed. These enhancements made SiPMs particularly suitable for LiDAR demanding high resolution, high sensitivity, and low power consumption. SiPMs have since been used in various applications, including autonomous driving, industrial measurement, and environmental monitoring.

Looking ahead to 2030–2040, SiPMs are anticipated to achieve further performance improvements, with lower noise levels, a broader spectral response range, and a higher operating temperature range. These advancements will enable SiPMs to handle more complex and demanding LiDAR tasks, such as 3D imaging, target identification, and remote detection. SiPMs are projected to maintain a pivotal role in intelligent transportation, robotic navigation, and military applications.

## 7. Application Cases

### 7.1. Railway Foreign Object Intrusion Monitoring in Tunnel Entrance and Subgrade near the Mountain

The environmental safety of train operation has emerged as a crucial issue in the safe operation of railway, which is predominantly manifested in the frequent occurrence of natural disasters along the route and the security risks in the surrounding environment [119]. Once a disaster strikes, it will severely disrupt the train operation sequence, endanger the safety of passengers’ lives and property, as well as social stability. Foreign objects such as landslides and debris flows will pose a significant threat to the safety of train operation [120].

The tunnel traverses the mountain. Climbers typically enter the tunnel at its entrance to avoid extreme weather and traverse the mountain. Intruders exhibit behavior characterized by wandering and prowling at the tunnel entrance. The tunnel mouth, cut, and subgrade section near the mountain are prone to rockfall, which can be caused by mountain collapse, falling trees, vegetation, or landslides due to weather. Should such events occur, they could bury the railway line, leading to severe accidents and complicating rescue efforts. Therefore, it is crucial to prevent personnel and foreign object intrusion at the tunnel mouth, cutting, and embankment section near the mountain to ensure safe railway operation.

**Figure 24:**
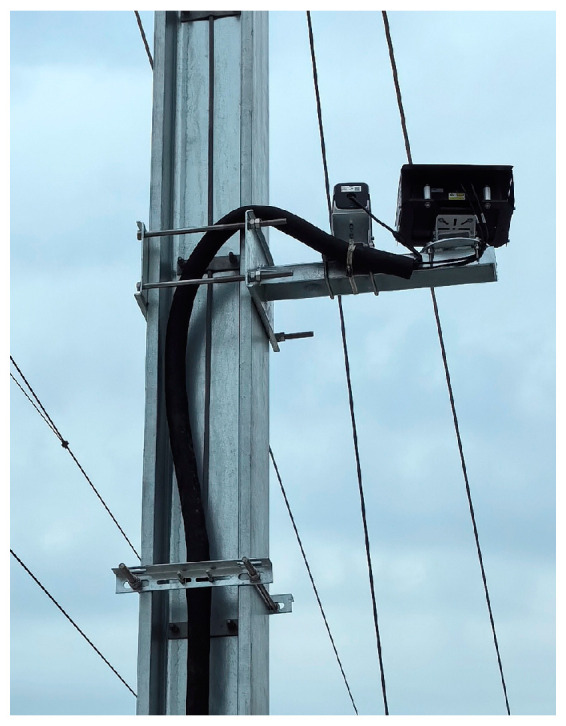
Railway foreign object monitoring equipment based on LiDAR and video fusion.

Currently, various techniques exist for perimeter protection of high-speed railways, but each individual technique faces challenges such as high false positive rates, high false negative rates, and elevated overall costs. LiDAR features high frequency, short wavelength, long-range, high-speed resolution, and precise positioning. In addition to generating clear 3D images to obtain accurate position information, LiDAR can also distinguish different target materials based on the reflectivity of the laser signal. Video surveillance is an active detection method that can effectively complement other technical systems. It offers a large amount of image data, strong intuitiveness, integrity and authenticity of recorded information, and robust real-time capabilities, providing the most accurate and immediate judgment of abnormal situations and their effects. The railway foreign object intrusion monitoring equipment based on LiDAR and video fusion is shown in Figure 24.

### 7.2. Geographic Information Systems (GIS) and Terrain Mapping

LiDAR has revolutionized GIS by enabling the creation of high-resolution digital elevation models (DEMs). These models accurately represent the Earth’s surface, providing critical data for terrain mapping. LiDAR’s precise elevation measurements allow for detailed analysis of topographical features, improving flood risk assessments and land-use planning [121].

In vegetation mapping, LiDAR penetrates through canopies to provide detailed data on forest structure, including canopy heights and ground surface. This capability is essential for monitoring forest ecosystems, assessing biomass, and evaluating habitat quality, contributing to ecological research and conservation efforts [122].

LiDAR also plays a key role in urban planning, providing detailed 3D models of infrastructure. These models help assess building heights, monitor land use changes, and optimize transportation networks, facilitating more effective urban development [123].

### 7.3. Autonomous Driving

**Figure 25:**
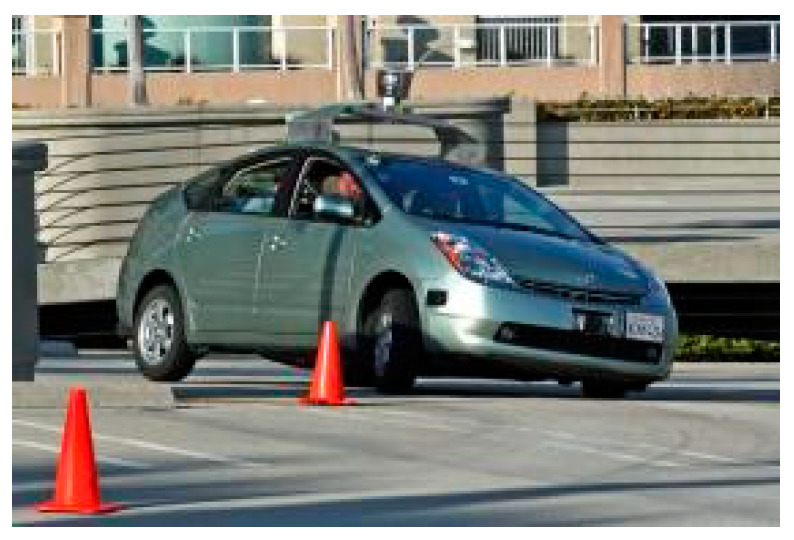
Google driverless car modified with Toyota.

Waymo, a pioneering program by Google, has been at the forefront of autonomous driving, with LiDAR as a key component. Initially launched in 2009 as Google’s “self-driving car project”, Waymo was led by Sebastian Thrun, known for his contributions to Google Street View. During the project’s early period from 2009 to 2012, the Waymo team focused on refining and validating autonomous driving [124]. They used a modified Toyota Prius for a test drive on public roads in California, as shown in Figure 25.

**Figure 26:**
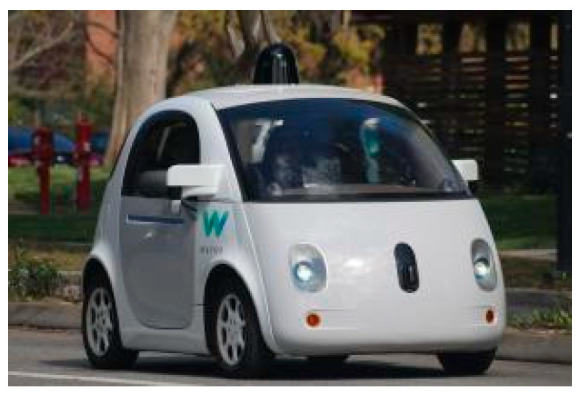
Waymo unmanned car.

By 2012, Waymo had surpassed 300,000 miles in autonomous testing. Between 2013 and 2015, Waymo introduced a fully autonomous vehicle, relying solely on self-driving technology for control, as shown in Figure 26. During this period, Waymo achieved significant technological advancements, particularly in enhancing its LiDAR system.

In 2017, Waymo launched its public pilot in Arizona, giving local residents access to its autonomous driving service. This was followed by the commercial launch of Waymo One in December 2018, offering ride-hailing services with autonomous vehicles to the general public. Over the past few years, Waymo has broadened its scope, collaborating to enhance its technology and expand services. These include partnerships with automotive giants like Chrysler, Volvo, and Nissan to advance autonomous driving solutions.

While Waymo has been a key player, many other companies have also developed advanced LiDAR systems for autonomous driving and ADAS (Advanced Driver Assistance Systems). Companies like Hesai, Ouster, Robosense, Luminar, Innoviz, and Aeye have introduced cutting-edge LiDAR solutions, contributing to significant advancements in the field. For instance, Luminar is known for its long-range LiDAR technology, which has been integrated into several autonomous vehicle platforms [125]. Innoviz offers solid-state LiDAR technology, focusing on reducing costs and increasing reliability for mass production [126]. Hesai and Robosense are leading LiDAR providers in China, with high-resolution, cost-effective solutions tailored for various applications, including self-driving cars and robotics [127]. Ouster has developed digital LiDAR technology that enhances resolution and reliability [128], while Aeye focuses on adaptive LiDAR systems that dynamically adjust based on driving conditions [129].

Waymo’s LiDAR stands out for its high resolution and expansive FOV, enabling the precise detection of minute details like traffic cones from several hundred meters. In urban environments, LiDAR helps identify and classify a diverse array of objects, both static and dynamic. On highways, it can gauge the speed and position of other vehicles, facilitating real-time adjustments to ensure safety. Waymo’s LiDAR emphasizes performance under challenging weather and lighting conditions. It is designed to discern raindrops or snowflakes from other critical objects, especially in adverse weather.

Safety is paramount in Waymo’s autonomous driving initiative. Its LiDAR provides precise data that complements inputs from various sensors. Sophisticated algorithms integrate and analyze this data, enhancing the vehicle’s environmental perception and enabling preemptive responses to traffic dynamics, mitigating risks.

Waymo’s project exemplifies the transformative potential of LiDAR in autonomous transportation, underscoring its pivotal role in enhancing road safety and advancing transportation evolution. As LiDAR technology advances and becomes more cost-effective, its integration into the autonomous driving sector is expected to increase, with companies like Hesai, Ouster, Robosense, Luminar, Innoviz, and Aeye continuing to play significant roles in this transformation, promising profound societal impacts.

### 7.4. Other Fields

In addition to autonomous vehicles, LiDAR technology is widely utilized across various platforms such as satellites, airplanes, ships, and drones, each designed for different operational requirements. Satellite-based LiDAR systems, for instance, are often used for large-scale Earth observation and atmospheric monitoring, providing valuable data for climate studies and land mapping. These systems require high energy efficiency and resilience to space conditions, with key components such as high-power lasers and sensitive detectors optimized for long-range sensing [130]. Airborne LiDAR, typically deployed on airplanes, is frequently used for topographic mapping and forestry management, where precision in capturing terrain elevation and vegetation structure is critical [131]. In contrast, ship-based LiDAR systems are often designed for maritime applications, such as mapping the seafloor or ensuring safe navigation through complex environments [132]. UAV-mounted LiDAR is gaining popularity in areas like precision agriculture, where lightweight and compact systems are necessary for drones to efficiently map farmland or monitor environmental conditions [133]. The design and performance metrics, including range, resolution, and power consumption, vary significantly depending on the specific application and platform, reflecting the diversity of LiDAR technology across multiple sectors.

## 8. Technical Challenges and Trends

In the evolution of LiDAR technology, several key trends are shaping its future. Solid-state LiDAR represents a significant advancement, as it eliminates moving parts, thus enhancing the system’s durability, reliability, and potential for miniaturization. This approach also contributes to cost-effectiveness, making LiDAR more accessible across various industries. Additionally, high integration is anticipated, where LiDAR systems will become more compact, with reduced size and weight, facilitating easier incorporation into devices such as smartphones and autonomous vehicles.

LiDAR technology is also expected to improve in terms of detection range and resolution, driven by advancements in laser emission power, detector sensitivity, and data processing algorithms. These developments will significantly enhance the system’s ability to detect objects at greater distances and with higher precision. Moreover, the adoption of multi-wavelength [134] capabilities and diverse scanning modes will enable LiDAR to adapt to different environmental conditions, making it more versatile and effective in various mission-critical scenarios.

Another promising trend is the integration of deep learning [135] and AI algorithms [136] into LiDAR systems, particularly in applications such as autonomous driving and long-range detection. These algorithms can greatly improve target detection, classification, and tracking, offering more sophisticated sensing capabilities. Alongside these advancements, the reduction in LiDAR costs is expected due to innovations in manufacturing processes, which will increase its deployment in consumer devices like smartphones [137], cameras [138], and drones [139].

Finally, multisensor fusion [140] is becoming increasingly important, where LiDAR data is combined with other sensor technologies, such as millimeter-wave radar and vidicons. This fusion provides a more comprehensive and accurate sensing solution, especially in complex and dynamic environments.

## 9. Conclusions

LiDAR can measure objects and environments with high precision, providing accurate 3D spatial data for various applications. LiDAR is crucial for enabling environmental sensing in railway foreign object intrusion detection, autonomous driving, and drones. Additionally, LiDAR has extensive applications in topographic mapping, archaeology, forestry, intelligent transportation, and robotics. Despite remarkable progress and widespread adoption, LiDAR’s future faces numerous challenges and opportunities.

Firstly, reducing costs is pivotal for realizing LiDAR’s wider commercial applications. High costs currently limit its adoption in the consumer market. Developing more cost-effective LiDAR is a significant trend. Secondly, maintaining measurement accuracy and reliability under complex environments, such as adverse weather and glare interference, is a significant challenge. Research into signal processing algorithms, improvements in laser transmission and reception techniques, and the use of multi-sensor fusion techniques can enhance LiDAR performance in complex environments. Furthermore, with advancements in AI and deep learning, effectively analyzing and processing the large volumes of point cloud data generated by LiDAR has become another major research focus. Developing more efficient data analysis and processing tools can accelerate the extraction of valuable information from LiDAR data, enhancing the system’s overall efficiency and application value. Lastly, interdisciplinary collaboration will be a critical driving force for advancing LiDAR technology and applications. Integrating professionals’ expertise in fields like optics, electrical engineering, computer science, and AI can foster innovation and expand LiDAR technology applications. For instance, combining LiDAR with autonomous driving and drone can enhance existing transportation systems and enable new service models like automated courier delivery, disaster monitoring, and emergency response.

In conclusion, LiDAR is rapidly developing, and its future applications appear promising; however, a series of technical and market challenges must be addressed. Through continuous research, innovation, and cross-disciplinary collaboration, LiDAR is set to play a pivotal role in various fields, bringing substantial value and transformation to society.

## Data Availability

Not applicable.
